# Haematological parameters and lipid profile abnormalities among patients with Type-2 diabetes mellitus in Ghana

**DOI:** 10.1186/s12944-018-0926-y

**Published:** 2018-12-13

**Authors:** Samuel Antwi-Baffour, Ransford Kyeremeh, Samuel Owusu Boateng, Lawrence Annison, Mahmood Abdulai Seidu

**Affiliations:** 1 Department of Medical Laboratory Sciences, School of Biomedical and Allied Health Sciences, College of Health Sciences, University of Ghana, P. O. Box KB 143, Korle-Bu, Accra, Ghana; 2 Department of Medical Laboratory Sciences, School of Allied Health Sciences, Narh-Bita College, Tema, Ghana

## Abstract

**Background:**

Diabetes mellitus is a non-infectious disease that has a high prevalence worldwide. Altered level of many haematological parameters have been observed in patients with diabetes. The levels of lipids are also affected in diabetes by many factors since carbohydrate metabolism affect lipid metabolism. So far, very little work has been done linking haematological parameters and lipid profile in diabetics. The purpose of this study was therefore to evaluate the haematological parameters and lipid profiles of patients with type-2 diabetes and to correlate the results.

**Method:**

Three hundred and four (304) patients with type-2 diabetes with an age range of 28 to 70 years (171 males and 133 females) were recruited. About 5 ml of venous blood samples were collected from each participant after an overnight fast. A part of the blood samples was used to determine the lipid profile parameters and the other parts for the haematological parameters. The Statistical Package for Social Science (SPSS) version 21.0 and Microsoft office excel (2010) for windows were used for the statistical analysis of the data. Pearson’s correlation were performed between haematological and lipid parameters. Significance was set at *p* < 0.05.

**Results:**

The means and standard deviation of all the lipid parameters except TC showed significant difference in both males and females. There was also proportional increment in LDL-C (in males), LDL-C and Triglycerides (in females) as the age of participants increased and the ratio of TC/HDL was higher in males. There was also significant difference in all of the haematological parameters between the male and female populations. Further, a strong, significant positive correlation between RBC and lymphocytes and lipid parameters was observed. However, the correlation between platelets, haematocrit and haemoglobin and the lipid parameters was negatively significant.

**Conclusion:**

From the results obtained, it can be concluded that there is significant difference in lipid parameters between male and female diabetic patients. Levels of LDL-C and Triglycerides increased as the age of participants increased and the male population showed increased risk for coronary disease. Almost all of the haematological parameters examined differed significantly between the sexes. There was also, both strong positive and negative correlations between the haematological parameters and the lipid profiles.

## Introduction

Diabetes mellitus (DM) is a non-communicable disease or carbohydrate metabolism disorder which results in increase in blood glucose level (hyperglycaemia) [1]. It is caused by the absence of insulin secretion due to either the progressive or marked inability of the β-Langerhans islet cells of the pancreas to produce insulin or due to defects in insulin uptake in the peripheral tissue (insulin resistance) [2]. DM is broadly classified under two categories - type 1 and type 2 diabetes [2]. Type 1 diabetes occurs most commonly in children, but it can sometimes also appear in adult age groups, particularly those in their late thirties and early forties [3]. The major factor in the pathophysiology of type 1 diabetes is considered to be autoimmunity [4]. Type 2 diabetes on the other hand has a different pathophysiology and etiology as compared to type 1 diabetes. Type 2 diabetes is described as a combination of low amounts of insulin production from pancreatic β-cells and peripheral insulin resistance [5]. Insulin resistance leads to elevated fatty acids in the plasma, causing decreased glucose transport into the muscle cells as well as increased fat breakdown, subsequently leading to elevated hepatic glucose production [5, 6]. Insulin resistance and pancreatic β-cell dysfunction must occur simultaneously for type 2 diabetes to develop [6].

Diabetes mellitus has increasing prevalence worldwide with certain ethnic and racial groups of Asia and Africa at a greater risk [7]. Poorly controlled diabetes leads to various complications such as nephropathy, retinopathy, neuropathy and oxidative stress causing oxidative damage to tissues and cells [8, 9]. The overall temporal burden of hyperglycemia is responsible for DM complications and adverse outcomes [10]. Patients with type 2 DM have increased risk of cardiovascular disease (CVD) related with atherogenic dyslipidemia, coronary artery disease, and myocardial infarction [9, 10]. The persistent hyperglycemia in DM results in disturbances in cellular metabolism due to increased production of reactive oxygen species (ROS) and non-enzymatic glycation of many macromolecules, which lead to changes in cellular structure and function and formation of advanced glycation end products [11]. The formation of advanced glycation end products enhances metabolic disturbances and also increases reactive oxygen species production via interaction with the specific receptor for the advanced glycation end product [12]. This causes changes in structure and biophysical properties of the basement membrane which further causes changes in permeability and vasodilatation of blood vessels [13].

Patients with diabetes mellitus show a significant derangement in various haematological parameters [14]. In fact, several haematological changes affecting the red blood cells (RBCs), white blood cells (WBCs), platelet and the coagulation factors are shown to be directly associated with DM [15, 16]. Systematic review and meta-analysis of cross-sectional and prospective studies have shown that the number of peripheral WBCs such as basophils, eosinophils and neutrophils increased with no change in the number of monocytes in patients with type-2 DM [16]. Furthermore, a study suggested that high platelet activity enhances vascular complications in DM patients and altered platelet morphology and function can be reflected as a factor for risk of microvascular and macrovascular diseases [17, 18]. Several studies have reported that increased platelet reactivation in patients with diabetes may confer less cardiovascular protection with antiplatelet therapy, particularly aspirin [19, 20]. In fact, it has already been demonstrated that insulin resistance and hyperinsulinemia are associated with the stimulation of erythroid progenitors and increased levels of inflammatory markers [21].

Other epidemiological studies have indicated a close relationship between the WBC count and components of metabolic syndrome [22]. These abnormalities have been shown to markedly increase blood viscosity that unfavourably affects the microcirculation, leading to microangiopathy [23]. It was revealed that higher WBC count, is one of the major components of inflammatory process that contributes to atherosclerotic progression and CVD [22, 24]. Haematological indices are therefore important indicators for the evaluation of variations in size, number and maturity of different blood cells and for the assessment and management of patients with DM [24, 25]. Therefore, this study which is aimed at determining haematological indices among type-2 DM patients will go a long way in assisting in their management.

It is known that many factors affect lipid levels in diabetes because carbohydrate metabolism directly affect lipid metabolism [26, 27]. Also insulin deficiency causes higher metabolization of free fatty acid and can cause disorder in lipid metabolism [28]. Lipid abnormalities have also been seen to play an important role in the increased vascular risk associated with type-2 DM [26, 27]. It is based on these facts that it was deemed fit to conduct this study to evaluate the lipid profile of patients with type-2 DM. A lipid profile is a direct measure of three blood components namely; total cholesterol (TC), triglycerides and high density lipoproteins cholesterol (HDL-C) [29]. There are components such as low density lipoprotein cholesterol (LDL-C) and very low density lipoprotein cholesterol (VLDL-C) that can also be derived from the direct measurements [29]. The burden of cardiovascular disease or coronary heart diseases in the world is enormous and growing and the majority of those affected are in developing countries [30]. Certain aspects of a person’s lifestyle including diet, level of physical activity, level of diabetes control and smoking status may affect lipid profile [31, 32].

The high incidence of atherosclerosis in elderly people suggests that age may be among the factors that affect lipid metabolism; hence putting elderly subjects at risk of developing cardiovascular or coronary heart diseases [33]. It was estimated in 2002 that, 29% of death worldwide (16.7 million deaths) were due to CVD [34]. Uncontrolled dyslipidemia also leads to various medical complications [35]. An increase in the incidence of coronary heart disease risk has commonly been reported in postmenopausal women [33]. The incidence of chronic heart disease (CHD) is much lower in young women than in men of the same age, up to the age of 65 years [36]. However, after the age 65, the risk equalizes for both sexes [36]. This has led to the popular misconception that cardiovascular disease is a disease of men, and is relatively rare in women but cholesterol levels tend to rise with age in both males and females [37]. There are a lot of studies comparing haematological parameters in patients with diabetes and others looking at lipids in diabetics but there is paucity in studies comparing both parameters in patients with diabetes. The aim of this study was therefore to evaluate the haematological parameters and lipid profiles of patients with type-2 diabetes and to correlate the results. It is believed this study will uplift awareness for the need of both haematological and lipid analysis in patients with diabetes so the necessary steps can be taken to optimize their management.

## Methods

### Study design

The study was a cross sectional study which was conducted between January and December 2017.

### Study setting

The study was conducted at the Korle-bu Teaching Hospital, Accra, Ghana.

### Characteristics of study participants

The study population was made up of three hundred and four (304) patients with type-2 diabetes with an age range of 28 to 70 years. There were 171 males and 133 females. All the participants said they practiced healthy eating which include the type of food they ate, how much they ate and the combinations of food types they ate. They also exercised regularly where possible and were not smoking or drinking alcoholic beverages. Finally they had regular check-ups by attending the diabetic clinic regularly. The population was stratified by age and gender into five (5) strata; 28–34 years, 35–40 years, 41–50 years, 51–60 years, 61–70 years.

### Sampling procedure

#### Questionnaire-based data collection

Data were collected through the use of a structured questionnaire. The information collected included the participant‘s name, age, sex, diabetes status, the intake of lipid lowering drugs and haematinics.

#### Biochemical measurements (lipid profile)

The biochemical measurements made were triglycerides, total cholesterol (TC), high density lipoprotein cholesterol (HDL-C) and low density lipoprotein cholesterol (LDL-C) levels in blood. About 5 ml of venous blood samples were collected from each participant after an overnight fast. A part of the venous blood samples collected were dispensed into serum separating tubes and allowed to clot. They were then centrifuged at 3000 rpm for 10 min at room temperature. Using standard laboratory practice, Triglycerides, TC, HDL-C and LDL-C were determined directly or samples were stored for analyses later.

#### Biochemical measurements procedure

The lipid profile parameters were determined using ELITech chemistry reagents kit from ELITech Group Clinical Systems (Paris, France). The cholesterol reagent kit with product code (SL) was used for cholesterol determination, the HDL-C reagent kit with product code (HDL SL 2G) was used for HDL-C determination and LDL-C precipitation and triglycerides reagent kit product code (MONO SL NEW) was used to determine LDL-C and triglycerides. The instrument used was the Mindray B-300 chemistry analyzer manufactured by Shenzhen Mindray Bio-Medical Electronics Company, Limited. The procedures of work and preparation of the working reagents were done as described by the manufacturer.

#### Diagnostic criteria

Dyslipidemia was considered in adult when total cholesterol level was ≥5.2 mmol/L, triglyceride level was ≥1.58 mmol/L, LDL-cholesterol level was ≥3.8 mmol/L and if HDL-cholesterol level was < 0.9 mmol/L according to established reference interval by the korle-bu Teaching Hospital central laboratory [38].

#### Haematological (FBC) analysis

Full blood count comprising red cell count, Hb, white cell count and differentials, platelets as well as Hb indices were determined from the remaining whole blood that was placed in EDTA test tubes using ABX Micros 60 Haematology Analyzer (Horiba-ABX, Montpellier, France). Thin blood film was prepared and stained using Leishman stain for morphologic assessment of the red blood cells. The stained films were examined under the light microscope using × 40 objectives to select a good area for examination and then a drop of oil placed on the film and examined with the × 100 objective [39].

### Data analysis

Data was collected using notebooks and transferred to a computer and kept confidential. They were later entered into Microsoft Word and analysed using Statistical Package for Social Sciences (SPSS, Version 21.0) and Microsoft office excel (2010) for windows. Normally distributed data were analyzed using independent sample t-test and expressed as Mean ± SD. Pearson’s correlation was used to determine the correlation between the obtained haematological parameters and lipid parameters. A *p*-value of < 0.05 was considered statistically significant.

## Results

The total number of individuals recruited into the study was 304 comprising of 171 (56%) males and 133 (44%) females. The average age of males was 45.8 ± 14.2 years and that of females was 46.0 ± 14.3 years. The means and standard deviation of all the lipid profile parameters of the participants were analyzed and it turned out that all the parameters except total cholesterol showed significant difference in both males and females (Table 1).

**Table 1:** A table of means and standard deviations of serum lipids measurements in both male and the female participants

Parameters	Males *N* = 171 Mean ± SD	Females *N* = 133 Mean ± SD	P-value
Age (years)	45.8 ± 14.2	46.0 ± 14.3	0.000
Total Cholesterol (mmol/L)	5.00 ± 1.1	5.2 ± 1.2	0.843
HDL Cholesterol (mmol/L)	1.5 ± 0.5	1.8 ± 0.7	0.000
LDL Cholesterol (mmol/L)	3.2 ± 1.1	3.2 ± 1.2	0.000
Triglycerides (mmol/L)	1.2 ± 0.6	1.1 ± 0.6	0.000

The various lipid parameters were analyzed against the different age groups among the male participants. It was realized that age group 61–70 had the highest TC (5.5 ± 1.3 mmol/L) and LDL-C (3.7 ± 1.4 mmol/L) level compared to the other age groups. Also, there was proportional increment in LDL-C as the age of the participants increased - a pattern that was however not seen in the other parameters (Table 2).

**Table 2:** A table of means and standard deviations of serum lipid measurements in studied age groups among the male participants

Age group	TC(mmol/L) Mean ± SD	HDL-C(mmol/L) Mean ± SD	LDL-C(mmol/L) Mean ± SD	TG(mmol/L) Mean ± SD
28–34 Years	4.6 ± 0.6	1.6 ± 0.5	2.8 ± 0.9	0.9 ± 0.2
35–40 Years	4.7 ± 0.7	1.4 ± 0.5	3.1 ± 0.8	1.1 ± 0.6
41–50 Years	5.2 ± 1.2	1.7 ± 0.6	3.2 ± 1.1	1.4 ± 0.8
51–60 Years	5.0 ± 1.3	1.4 ± 0.5	3.3 ± 1.2	1.3 ± 0.5
61–70 Years	5.5 ± 1.3	1.5 ± 0.5	3.7 ± 1.4	1.2 ± 0.5

Again, the individual lipid parameters were analyzed for the female participants. Here it was seen that the LDL-C and triglycerides levels increased as the participant age increased. Also, the TC among age group 28–34 years in females was the lowest as compared to the other studied age groups. However, age group 61–70 years had the highest value of HDL-C and LDL-C (Table 3).

**Table 3:** A table of means and standard deviations of serum lipids measurements in studied age groups among the female participants

Age group	TC(mmol/L) Mean ± SD	HDL-C(mmol/L) Mean ± SD	LDL-C(mmol/L) Mean ± SD	TG(mmol/L) Mean ± SD
28–34 Years	4.8 ± 0.8	2.0 ± 0.4	2.2 ± 0.8	0.8 ± 0.3
35–40 Years	5.0 ± 0.9	1.6 ± 0.4	2.9 ± 0.9	0.9 ± 0.4
41–50 Years	4.9 ± 0.8	1.6 ± 0.5	3.0 ± 0.8	1.2 ± 0.5
51–60 Years	5.8 ± 0.9	1.6 ± 0.5	3.8 ± 1.0	1.3 ± 0.6
61–70 Years	5.3 ± 1.9	2.3 ± 1.2	3.9 ± 1.4	1.4 ± 0.6

**Fig. 1:**
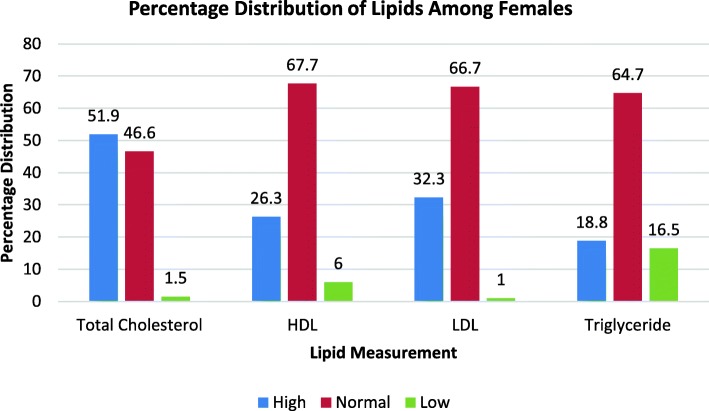
Percentage Distribution of Lipids among Female Participants

**Fig. 2:**
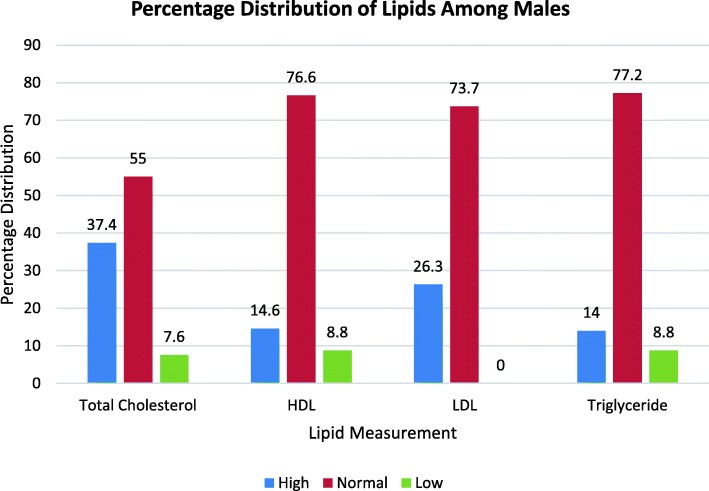
Percentage Distribution of Lipids among Male Participants

Furthermore, the percentage distribution of the lipid parameters into high, normal and low level categories was carried out among the participants. The average percentage of the participants that fell into each category was presented using a histogram. The outcome indicated that majority of the participants fell into the normal level category with all the parameters followed by high level category and then low level category for the female participants (Fig. 1). The analysis for the male participants followed similar pattern except that here the numbers that fell into the low level category were quiet low and the difference between normal and high levels of the parameters were significant (Fig. 2).

The ratio of TC/HDL cholesterol as an indicator of coronary risk factor was also calculated for both male and female participants and their mean was found to be 3.7 ± 1.7 mmol/L in males and 3.3 ± 1.5 mmol/L in females. The ratio was therefore high in males than females and subsequently showed a significant difference as far as both sexes are concerned (*P* = 0.000) (Table 4).

**Table 4:** A table of means and standard deviations of TC/HDL ratio in the studied age groups for both males and females

Age groups	Males TC/HDL ratio (mmol/L) Mean ± SD	Females TC/HDL ratio (mmol/L) Mean ± SD	P-value
20–30 years	3.3 ± 1.5	2.5 ± 0.5	0.061
31–40 years	3.8 ± 2.0	3.3 ± 1.2	0.314
41–50 years	3.5 ± 1.5	3.5 ± 1.3	0.521
51–60 years	3.9 ± 1.3	4.0 ± 1.9	0.000
61–70 years	4.3 ± 1.9	3.2 ± 1.8	0.000

From the table, it can be seen that the level of TC/HDL cholesterol ratio was high in males of age group 20–30 years and 31–40 years as compared to females of the same age group. The ratio (3.5 ± 1.5; 3.5 ± 1.3) for 41–50 years group of both sexes was almost the same. There was however a significant difference in TC/HDL ratio between the age groups of 51–60 years and 61–70 years in males and females (P = 0.000).

An independent sample t-test was conducted to investigate the haematological parameters of the participants across gender and there was significant difference in almost all of the parameters between the males and females (Table 5).

**Table 5:** A table highlighting the haematological indices of study population

Parameter	Females	Males	t	P-value
WBC	5.73 ± 0.21	8.19 ± 5.87	−2.405	0.022*
RBC	3.98 ± 0.61	4.57 ± 0.61	−4.567	0.000*
Haemoglobin	10.63 ± 0.79	11.80 ± 1.10	−9.328	0.000*
HCT	31.96 ± 2.36	35.44 ± 3.28	−9.631	0.000*
MCV	75.59 ± 5.57	85.35 ± 3.94	−4.928	0.000*
Platelets	224.56 ± 18.71	275.85 ± 28.53	−8.767	0.000*
Lymphocyte	1.49 ± 0.15	1.63 ± 0.11	4.280	0.000*
Neutrophils	4.11 ± 0.54	5.80 ± 0.41	11.325	0.000*

*WBC* white blood cell, *RBC* red blood cell, *HCT* haematocrit, *MCV* mean cell volume. Values are presented as mean ± standard deviation. P < 0.05 is considered significant. *mean difference is statistically significant,

The Pearson-Moment-*r* correlation test was employed to investigate the existence of any significant relationship between haematological parameters and the different lipid profile parameters among the participants. There was a strong, positive correlation between RBC and lymphocytes and the different lipid parameters, which was also significant [*p* = 0.003 and *p* = 0.002 respectively].

Also, there was a strong negative correlation between platelets, WBC, HCT, Haemoglobin, MCV and Neutrophils. Whilst with Platelets, Haemoglobin and HCT the correlation was statically significant [p = 0.003, p = 0.003 and *p* = 0.020 respectively], the correlation of WBC, MCV and Neutrophils did not show significance [*P* = 287, *p* = 0.720 and *p* = 0.745 respectively] (Table 6).

**Table 6:** A table showing the correlation between haematological parameters and serum lipids measurements using Pearson moment-r correlation

Parameter	Total cholesterol		HDL		LDL		Triglyceride	
	r	*P*-*value*	r	*P*-*value*	r	*P*-*value*	r	*P*-*value*
Platelets	− 0.496	0.003*	− 0.478	0.004*	− 0.522	0.002*	−0.488	0.003*
WBC	−0.177	0.318	−0.165	0.296	−0.169	0.321	−0.178	0.287
RBC	0.526	0.001*	0.498	0.021*	0.532	0.002*	0.518	0.003*
Haemoglobin	−0.514	0.002*	−0.510	0.010*	−0.518	0.004*	−0.499	0.003*
HCT	−0.525	0.001*	−0.521	0.002*	−0.530	0.010*	−0.518	0.020*
MCV	−0.043	0.808	−0.039	0.718	−0.041	0.691	−0.050	0.720
Lymphocyte	0.432	0.005*	0.442	0.005*	0.401	0.003*	0.512	0.002*
Neutrophils	−0.028	0.873	− 0.018	0.773	− 0.030	0.678	− 0.029	0.745

*WBC* white blood cell, *RBC* red blood cell, *HCT* haematocrit, *MCV* mean cell volume. *mean difference is statistically significant where P < 0.05 is considered significant

## Discussion

From the data obtained, the means and standard deviation of all the lipid parameters except total cholesterol (TC) showed significant difference in both males and females. In fact, the mean total cholesterol was high in females than males (Table 1). The higher TC in women may be due to higher sex hormone, particularly E2 in females and its effect on lipid metabolism [40]. This finding was similar to a study presented on the distributions of blood lipids profile for a geographically defined cohort of rural elderly Iowans which demonstrated a higher level of TC in women compared to men [41]. Another research code named “the Bronx Aging Study” that was done in the same year to assess risk factors for the development of dementia, coronary and cerebrovascular diseases in elderly people again demonstrated significantly higher total cholesterol in women compared to men [42]. The finding of high cholesterol in females than males was however contrary to a finding made in a study by Adediran and colleagues (2012), where 39.7% of males and 54.5% of the females had low cholesterol values [43].

With regards to HDL-C, it was seen that the females had higher mean value than the males and this was so because from puberty on, women tend to have higher HDL-C levels than men due to the production of estrogen [44]. Again, looking at the LDL-C results, the mean values were almost the same between both sexes. This was expected based on the average age of both sexes in the study for even though young women tend to have lower LDL-C levels than young men this changes after menopause. After menopause, the level of LDL-C in women tends to increase in equal measure to men as a result of lack of estrogen. Estrogen increases hepatic cell surface LDL-C receptors and consequently rapid clearance of LDL-C particles in premenopausal women. However, in menopausal state this clearance is reduced due to limited estrogen production [40, 45]. Now, looking at the values obtained for triglycerides, the males had higher value than the females and this follows the assertion that men tend to have higher triglycerides than women [46].

Also, the various lipid parameters were analyzed against the different age groups and gender among the participants. From the male population (Table 2), it was seen that there was a rise in total cholesterol values from age group 28 – 34 yrs. up to the 41 – 50 yrs. group. Then the value dropped a bit within the age group of 51 – 60 yrs. and thereafter was raised in the last age group of 61- 70 yrs. Still with the HDL-C values for the male population, the age group of 35 – 40 yrs. had a slightly lower value against the first age group. Then the value picked up in the following age group (41 – 50 yrs) and then dropped again within the age group of 51 – 60 yrs. and subsequently increased slightly within the last age group. When it came to LDL-C, a marginal but exponential increase in values from the lower age group (28 – 30 yrs) to the highest (61 – 70 yrs) was seen. The triglycerides also showed a similar pattern up to the age group of 51- 60 yrs. and dropped slightly within the age group of 61 – 70 yrs.

Now from the female population (Table 3), total cholesterol was normal for the age group of 28 – 34 yrs., it then rose slightly within the next age group of 35 – 40 yrs. and from there it dropped slightly within age 41 – 50 yrs., increased again within age 51 – 60 yrs. and finally declined minutely within age 61 – 70 yrs. For HDL – C, a normal value was seen for age group 28 – 34 yrs. and then it reduced slightly and maintained the level for age group 35 – 40 yrs. up to 51 – 60 yrs. after which it increased slightly in the last age group. With LDL-C, there was exponential increase as the age increased and the same pattern was seen with the triglyceride results. This finding is in line with a study by Schaefer and colleagues in 1994 which postulated that, increased age was associated with higher plasma LDL-C, especially in women and was significantly higher in postmenopausal than in premenopausal women [47].

Again, the percentage distribution of the lipid parameters into high, normal and low level category was carried out among the participants. The outcome indicated that majority of the participants fell into the normal level category with all the parameters followed by the high level and then low level category for the female participants (Fig. 1). The analysis for the male participants follow similar pattern except that in their case the numbers that fell into the low level category were quiet low and the difference between normal and high levels of the parameters were significant (Fig. 2).

Furthermore, the ratio of TC/HDL cholesterol as an indicator of coronary risk factor was calculated for both male and female participants. It could be seen that the level of TC/HDL cholesterol ratio was high in males of age group 20–30 years and 31–40 years as compared to females of the same age group. The ratio (3.5 ± 1.5 and 3.5 ± 1.3) for 41–50 years group of both sexes was almost the same. There was however a significant difference in TC/HDL cholesterol ratio as far as males and females are concern in age group 51–60 years and 61–70 years (*P* = 0.000). According to American Heart Association, TC/HDL cholesterol ratio should ideally be ≤3.5 mmol/L and even though both males and females are at risk of developing CVD especially elderly ones, it is not the same in both subjects [48]. According to Maas and Appleman (2010), although women and men share most classic risk factors, the significance and the relative weighting of the factors are different and cardiovascular disease develops earlier in men than women [49].

The other part of the study looked at haematological parameters and an independent sample t-test was conducted to investigate the parameters across gender among the participants. It was seen that there was significant difference in almost all of the haematological parameters between the males and females. Also, when Pearson-Moment-*r* correlation test was employed to investigate the existence of any significant relationship between haematological parameters and the different lipid parameters among the participants, there was a strong, positive correlation between RBC and lymphocytes and the different lipid parameters which was also significant [*p* = 0.003 and *p* = 0.002 respectively]. Again, there was a strong negative correlation between platelets, WBC, HCT, Haemoglobin, MCV and Neutrophils. However, only Platelets, Haemoglobin and HCT showed statically significant correlation [p = 0.003, p = 0.003 and *p* = 0.020 respectively] whilst WBC, MCV and Neutrophils did not show significance [*p* = 287, *p* = 0.720 and *p* = 0.745 respectively] as seen from Table 6. The findings of this study is believed to be novel and we hope it will go a long way to assist in the management of patients with type-2 diabetes.

A limitation worthy of mention was the inability to repeat the tests for all the subjects at different time points, due to limited resources and time constraints.

## Conclusion

The outcome of the study indicates that there is significant difference in lipid parameters between males and females. Again, we saw proportional increment in LDL-C in males and LDL-C and Triglycerides in females as the age of participants increased. Furthermore, the coronary risk factor was higher in males than females and the difference was significant. With regards to haematological parameters, we saw significant difference in almost all of the haematological parameters between the male and female participants. There was also, both strong positive and negative correlations between the haematological parameters and the different lipid parameters. This study presents some interesting and novel findings which may be very important in the care and management of patients with type-2 diabetes.

## Abbreviations

CHD: Chronic Heart Disease
CVD: Cardiovascular Disease
DM: Diabetes Mellitus
EDTA: Ethylene Diamine-Tetra-Acetic Acid
Hb: Haemoglobin
HCT: Haematocrit
HDL-C: High Density Lipoprotein Cholesterol
LDL-C: Low Density Lipoprotein Cholesterol
MCV: Mean Cell Volume
RBC: Red Blood Cell
ROS: Reactive Oxygen Species
SD: Standard Deviation
SPSS: Statistical Package for Social Science
TC: Total Cholesterol
VLDL-C: Very Low Density Lipoprotein Cholesterol
WBC: White Blood Cells
WHO: World Health Organization
